# From sequence to activity: the *Hga*I-homologous restriction modification system RM.*Mho*VI of *Mycoplasma hominis*

**DOI:** 10.1186/s12866-025-04270-3

**Published:** 2025-08-25

**Authors:** Lars Vogelgsang, Manuel Dolgopolow-Schmidt, Azlan Nisar, Dana Bäcker, Alexander T. Dilthey, Birgit Henrich

**Affiliations:** Institute of Med. Microbiology and Hospital Hygiene, Heinrich-Heine-University Duesseldorf, Duesseldorf, Germany

**Keywords:** *hga*I, *Mycoplasma hominis*, RM system, methylation-sensitive restriction, Modified bases, 5mC, Dorado, Nanopore sequencing

## Abstract

Restriction-modification (RM) systems are widespread defense mechanisms in prokaryotes that protect the host from potentially harmful foreign DNA. They typically consist of a DNA methyltransferase (MTase), which methylates the host genome at an adenine (6 mA methylation) or cytosine (4mC or 5mC), and a restriction endonuclease (REase), which cleaves foreign, unmethylated DNA. In addition to the 2023 published family of 5mC-MTases, an *Hga*I-homolog RM system was detected in *Mycoplasma hominis* with the more rare constellation of two 5mC MTase genes, called RM.*Mho*VI. A qPCR screening of 239 randomly selected *M. hominis* isolates revealed a prevalence of the *Mho*VI-RM system of 12.97% (*n* = 31/239). Notably, in all tested *Mho*VI-positive isolates, the *Mho*VI-RM cassette localized between MHO_3110 and MHO_3120 and comprised an XRE-family transcriptional regulator gene in addition to the RM genes. Intra-species conservation of the encoded *Mho*VI-enzymes was high (> 99% identities), and inter-species conservation was the lowest compared to the eponymous species *Haemophilus gallinarum* (46.6% M1.*Mho*VI; 48.1% M2.*Mho*VI; 27.4% R.*Mho*VI). A polycistronic organization of the *Mho*VI-genes was strongly suspected due to the discovery of gene-overlapping mRNA regions. The MTases activity was demonstrated in RM.*Mho*VI positive *M. hominis* isolates by protection of genomic DNA from cleavage by the methylation-sensitive endonuclease *Hga*I; and bioinformatics analysis using the Dorado basecaller on the Oxford Nanopore sequenced genomes revealed methylation rates of the respective motifs, 5’-GA^m^CGC-3’/5’-G^m^CGTC-3’, above 95% in *Mho*VI-positives, with a higher methylation frequency of 5’-GA^m^CGC-3’ than 5’-G^m^CGTC-3 in most isolates. A final proof of *Mho*VI-RM representing an *Hga*I-RM-like methylation activity was demonstrated through expression and analysis of recombinant rM2.*Mho*VI in *E. coli*.

**Supplementary Information:**

The online version contains supplementary material available at 10.1186/s12866-025-04270-3.

## Background

*M. hominis* is a facultative pathogen of the human urogenital tract associated with local infections, such as pelvic inflammatory disease or bacterial vaginosis, and disseminated infections, like bacterial arthritis or preterm birth [1, 2]. The factors which render this cell wall-less mollicute from urogenital commensal to pathogen are not fully understood, but are hypothesized to result from its genetic heterogeneity, which derives from a strain-specific spectrum of variable lipoproteins, mobile genetic elements (MGE) and restriction modification (RM) systems [3].

In prokaryotes, RM systems function as the main defense mechanism against the invasion of foreign DNA by methylating specific sequence motifs of their own DNA by the DNA methyltransferase (MTase) and cutting the unmethylated motifs of the foreign DNA by the restriction endonuclease (REase) [4]. The acquisition of RM systems is mostly derived from horizontal gene transfer (HGT) as part of mobile genetic elements [5, 6]. Once integrated, they act as “selfish genetic elements” by increasing their prevalence in bacterial populations by exerting a form of genetic control through post segregational killing [7]. The loss of the MTase gene leads to cleavage of the unprotected host DNA by residual REase activity, thereby creating selective pressure to retain the RM system. After RM uptake, a controlled RM gene expression is essential to ensure that REase activity does not start earlier than methylation and thus lead to damage of the host DNA [8]. The transcriptional regulation of Type II RM systems, the most abundant subgroup of RM systems, is highly diverse. It ranges from polycistronic organization, where a single mRNA encodes all components (e.g., RM.*Sal*I in *Streptomyces albus*), to monocistronic mRNAs regulating each gene individually (e.g., RM.*Eco*RII in *E. coli*) [9, 10]. Complex RM systems, such as RM.*Eco*29kl involve fine-tuned regulation by multiple intrinsic promotors, while in others, like RM.*Ahd*I in *Aeromonas hydrophila*, the presence of small additional genes that encode transcriptional regulators has been described [11–14]. These regulators often belong to the XRE (xenobiotic response element) family, which is characterized by a DNA-binding helix-turn-helix (HTH) domain. The HTH domain is involved in promoter binding and regulation of respective gene expression and is also found in RM systems like RM.*Fok*I [15–17]. The controller (or C) proteins are the best-studied members of this family. As documented in REBASE, their genes are mostly positioned upstream of REase genes enabling the control of REase transcription, whereas the MTase genes are positioned on the complementary strand (see supplementary Tab. S2) [18–23].

RM systems are widely distributed among bacteria, with a vast diversity of types and specificities. Each RM system targets a specific motif. The motifs of type II RM systems are typically 4–6 bp sequences and mainly but, not always palindromic [24–26]. The MTase adds a methyl-group to either adenine (6 mA-methylation) or cytosine (5mC- or 4mC-methylation) of the motif, thereby protecting the motif against endonucleolytic digest by the REase [27]. The identification of the RM recognition motif is the basis of further investigation of the enzymatic activity of MTase and REase and their possible role in the organism.

Methylated DNA bases were detected in the early 1990 s by sequencing bisulfite-treated DNA [28]. Established methods, like methylation-sensitive-restriction (MSR), enable identification of methylated motifs to a certain degree [29, 30]. However, these methods fail to quantify methylation rates. By now, more flexible and universally applicable bioinformatical methods have been developed and allow identification of 4mC, 5mC and 6 mA modified bases with single-molecule real-time sequencing (SMRT) by Pacific Biosciences or Oxford Nanopore Sequencing Technology (ONT) [31, 32]. SMRT has long been considered the gold standard due to its relatively low error rate. However, recent advances in Oxford Nanopore Technology (ONT), including the introduction of R10 flow cells and updated chemistry, have significantly improved its accuracy [31].

In the REBASE database (http://rebase.neb.com), which is an extensive collection of information about restriction enzymes and DNA methyltransferases, three type II restriction enzymes have been deposited for *M. hominis*: the *Asu*I-homolog *Mho*I, the *Hha*I-homolog *Mho*2965 and the *Alu*I homolog *Mho*2111I. From our group, four additional type II RM systems of *M. hominis* have recently been described with homologies to RM.*Dpn*II (named RM.*Mho*II; recognizing the motif 5’-G**A**TC-3’), RM.*Sau3*AI (RM.*Mho*III; 5’-GAT**C**−3’), RM.*Hha*I (RM.*Mho*IV, which corresponds to *Mho*2965); 5’-G**C**GC-3’) and RM.*Eco4*7II (RM.*Mho*V; 5’-GGN**CC**−3’); with the methylated nucleotides underlined [30]. In this study, RM.*Mho*VI is characterized as a homolog of the *HgaI* RM system of *H. gallinarum*, which harbors two MTases - M1 and M2 - that methylate the complementary motif G^m^CGTC and GA^m^CGC, respectively [26].

## Methods

### Mycoplasma culturing and nucleic acid preparations

Clinical *M. hominis* strains were cultivated in arginine-medium and nucleic acids were prepared from *M. hominis* cultures in mid- to late logarithmic growth phase as described in detail previously [33]. Proteinase K digested cell lysate from 1 mL culture was used for qPCR analysis. Genomic DNA, isolated by the DNeasy Blood and Tissue kit (Qiagen, Hilden, Germany) [34], was used for methylation-sensitive restriction (MSR-) analysis and nanopore sequencing. RNA was prepared with the RNeasy Kit (Qiagen GmbH, Hilden, Germany) and cDNA was synthesized as published [35].

Concentration of isolated nucleic acids was measured by Qubit 4 Fluorometer (Invitrogen, Carlsbad, USA) and the quality was verified by NanoDrop 1000 Spectrophotometer (Thermo Fisher, Waltham, USA).

### qPCR

Oligonucleotides used in PCRs were designed using Probefinder (Roche Applied Science) (https://qpcr.probefinder.com) or PrimerSelect of DNASTAR (Madison, WI, USA). Primer sequences are listed in Table 1.

**Table 1:** Primers used

primer	sequence (5’−3’)	position
hitA_F	TTGAGGCACAGCAATAGC	439 269–439 286^1^
hitA_R	AAGGCTTAGGTAAGGAATGATTAG	439 204–439 227^1^
gap_F	GCAGGCTCAATATTTGACTCACT	660 140–660 162^1^
gap_R	GATGATTCATTGTCGTATCATGC	660 212–660 234^1^
lgt_F	TGAAATTGATTACGTCCAGGAA	420 553 − 420 574^1^
lgt_R	CCGAAACGAATTATTCCATAATAAAC	420 507 − 420 532^1^
upXRE_F1	CGACATATTTTATTAAAGACTTAAAACTTATT	497–528^2^
upXRE_F2	GATATGCCATGGTATTAATCAGTTAT	571–596^2^
XRE_F	GAGAATTGACCGGATTAGGAACR	649–671^2^
XRE_R	CTCCACCACATCTTCTATTTTACAATTT	743–770^2^
M1_R1	CTATTAAAATATAATTAAAATCATTATCTTCA	1224–1251^2^
M1_F	GAGTTCACACTGCACAACATATCTTAGTT	1451–1479^2^
M1_R2	TTTTTGGATAGAAGACAGGATTTTC	1513–1537^2^
M1_F2	ATGAAATTACTGGTGAAAGACTAAAAG	1538–1564^2^
M2_R1	TCTTCAATAATATCCATTACATAAAAAAC	2156–2184^2^
M2_F	GGATGGATCTCCAATTAAAGGTTTT	2596–2620^2^
M2_R2	CCACCCATATTGCCATTATTCA	2673–2694^2^
R_F	TTCGCTTTAGTCAAATGTGCTTTT	3113–3136^2^
R_R	AGGATGTGGATATTGCAAAGCAA	3245–3267^2^
downR_F	GTATAGTTGTTGCTCCACGTTTCTCAC	4172–4198^2^
downR_R1	GCTGTATTGATTATTGTTTTTTTCAT	4448–4473^2^
downR_R2	ACGACCTAACTCATAATTGAGCCAAG	4483–4508^2^

^1^Position in *M. hominis* PX1114 (acc.-no. CP032849.1)

^2^Position relative to the *mho*VI-gene cassette in PX1114 (pos. 391 805–386 982; see Fig. 1)

The qPCR assays were carried out in a total volume of 25 µl consisting of 1 × MesaGreen MasterMix, 5 mM MgCl_2_, Amperase, 300 nM of each primer and 2.5 µl of genomic DNA (1 ng/µl) or cDNA (0.8 ng/µl) solution, which was derived from 100 ng RNA [35]. Thermal cycling conditions were as follows: 1 cycle at 50 °C for 10 min, 1 cycle at 95 °C for 5 min followed by 35 cycles of 95 °C for 15 s and 60 °C for 1 min. PCR products are listed in Table 2.

**Table 2:** PCR-products

PCR	forward primer	reverse primer	length [bp]
XRE	XRE_F	XRE_R	122
M1	M1_F	M1_R2	87
M2	M2_F	M2_R2	99
R	R_F	R_R	155
A	upXRE_F2	M1_R1	681
B	M1_F2	M2_R1	647
C	M2_F	R_R	672
PI	upXRE_F2	XRE_R	200
PII	upXRE_F1	XRE_R	274
TI	downR_F	downR_R1	302
TII	downR_F	downR_R2	337

PCR products were differentiated from primer dimers with melting curve analysis by subsequent heating from 65 °C to 95 °C with an increment of 0.5 °C/15 s. Cycling, fluorescent data collection and analysis were carried out on a CFX-Cycler of BioRad Laboratories (Munich, Germany) according to the manufacturer’s instructions. Ct values of genomic DNA amplification were interpreted relative to the chromosomal *M. hominis*-specific *hit*A gene [36]. Transcript levels were calculated relative to the mean of two mycoplasma (housekeeping) genes, the glyceraldehyde-3-phosphate dehydrogenase (*gap*, also known as GAPDH) and the prolipoprotein diacylglycerol transferase *(lgt)*, which had formerly been shown to be unregulated [3, 35]. Ct-values > 27 were interpreted as negative. Larger regions of the *Mho*VI-gene cassette (> 500 bp) were amplified in 3-step qPCR with 35 cycles of 95 °C for 30 s, 60 °C for 30 s and 72 °C for 2 min.

### Cloning and expression of recombinant methyltransferases rM1 and rM2

The protein encoding regions of M1.*Mho*VI and M2.*Mho*VI of *Mycoplasma hominis* strain 16753VA were amplified by PCR using oligonucleotides (Metabion, Planegg, Germany) that change the mycoplasma TGA tryptophan codon to TGG (rM2) and add restriction sites (*Bam*HI and *Hin*DIII) up- and downstream of the open reading frames, respectively. rM2 fragments were fused by SOE (splicing by overlap extension)-PCR [37]. Amplicons were ligated in-frame into the expression vector pQE30 (Qiagen, Hilden, Germany) and plasmids were propagated in *E. coli* DH5α F’Iq. Sequence integrity of the inserts was analyzed by Sanger sequencing.

LB-medium (10 g/L bacto-tryptone, 5 g/L bacto-yeast extract, 5 g/L sodium chloride) containing 50 mg/L ampicillin and 0.02 mM IPTG (isopropylthio-P-D-galactoside) was inoculated with 1 mL of an overnight culture of the appropriate *E. coli* clone and incubated at 37 °C with vigorous shaking until an OD600 of 1.8 was reached. The cells were harvested by centrifugation (4500 x *g*, 20 min, 4 °C) and the cell sediments stored at −20 °C until use.

### Methylation sensitive restriction (MSR) analysis

Genomic DNA (0.75 µg) was digested with 1 U restriction enzyme *Hga*I (GACGCN_5/10_), *Dpn*I (G^m^A/TC) and *Mbo*I (GATC/N) in 15 µL for 1 h at 37 °C, followed by heat inactivation of the enzyme at 80 °C for 20 min and subsequent analysis on 0.8% agarose gels.

### Sanger-sequencing

For Sanger-sequencing, PCR-products were purified with “NucleoSpin Gel and PCR Clean-up” Kit from Macherey-Nagel (Dueren, Germany) and sequenced at the “Genomics & Transcriptomics Laboratory” of the Biological Medical Research Centre of the Heinrich-Heine University of Duesseldorf.

### Genome sequencing

Whole genome sequencing of clinical *M. hominis* strains and genetically modified *E. coli* was performed using Oxford Nanopore technology KIT (SQK-NBD114.24) according to the manufacturer’s instructions. The DNA libraries, generated by following the barcode ligation protocol (SQK-LSK109), were loaded on PromethION R10.4.1 flow cells and sequenced on the PromethION PC24 with the MinKNOW 23.11.7 software. Assembly of the nanopore long reads was conducted via Canu 2.1 (https://www.github.com/marbl/canu). Normalization of the genome sequences was done with respect to gene order and position in type strain PG21 (Acc.-Nr. FP236530.1) using Geneious Pro 5.5.8. Genome sequences are deposited at 10.17605/OSF.IO/3N4SY.

### Software based analyses

Geneious Pro 5.5.8 was used for multiple sequence alignments, generation of consensus sequences of overlapping PCR products and identification of open reading frames. BLASTP (https://www.blast.ncbi.nlm.nih.gov/Blast.cgi?PAGE=Proteins) was used for protein prediction and analysis. Prokaryotic promoters were calculated by ProPr 2.0 and Sapphire [38, 39]; transcription terminators by ARNold [40]. Multiple sequence alignments were calculated using Geneious Pro vers. 5.5.8 (Dotmatics, Boston, Madison, WI, USA). MegAlign 5.08 of the Lasergene software package (DNAStar, Madison, WI, USA) was used with default settings for phylogenetic tree construction.

### Calculation of methylation scores

FAST5 files of the Oxford Nanopore-derived reads that contain the methylation data underwent processing with the pod5 package (ver. 0.3.10) within the Miniconda3 24.3.0 environment to convert them to POD5 files. Methylation scores for 5-methylcytosine (5mC) were computed via the basecaller Dorado (ver. 0.5.1; https://github.com/nanoporetech/dorado), employing the basecalling model dna_r10.4.1_e8.2_400bps_sup@v4.2.0_5mC@v2 and using the respective Canu-assembled *M. hominis* genomes as reference. Conversion of BAM output files to BED files was done using the modbam2bed tool (ver. 0.10.0) to calculate the methylation frequencies of each cytosine. Positions of the internal cytosines in the *Mho*VI motifs (5’-GACGC-3’/5’-GCGTC-3’) were identified in genomes through JupyterLite (vers. 0.3.0; https://github.com/jupyter/notebook). The motif positions were than combined with the methylation frequencies to calculate the motif-specific methylation scores.

## Results

**Fig. 1:**
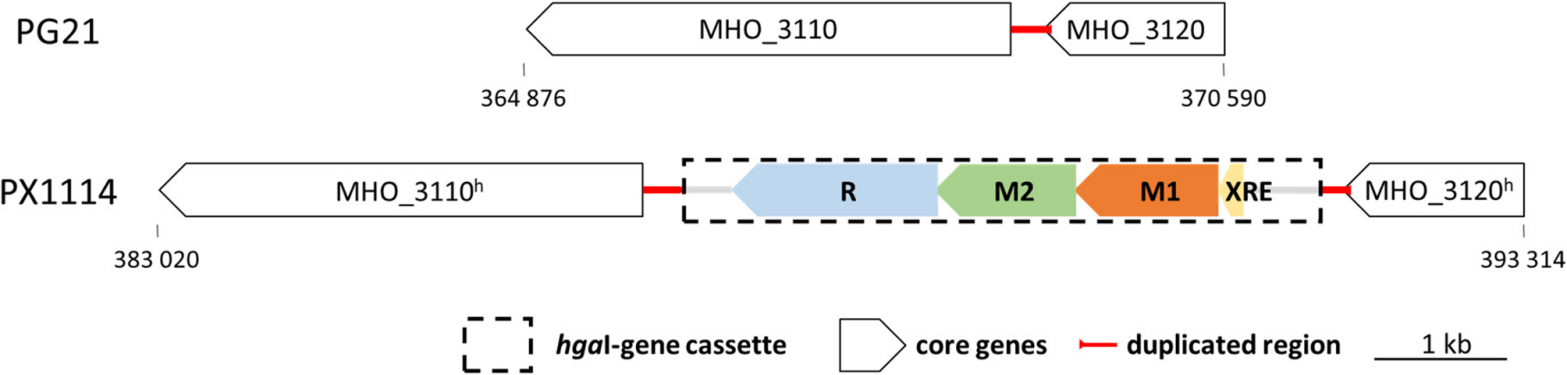
Scheme of the genomic locus of the *mho*VI-RM cassette. The *mho*VI-RM gene cassette (dashed square), schematically shown for isolate PX1114 (acc.-no. CP032849.1), encodes an XRE-transcriptional regulator (yellow), two MTases (M1.*Mho*VI (orange) & M2.*Mho*VI (green)) and a REase (R.*Mho*VI (blue)) and is positioned at an intraspecies-conserved integration site between the MHO_3110- and MHO_3120-homolog (^h^) core genes (with respect to the PG21 genome; white; acc.-no. FP236530.1) in reverse complementary direction. Integration caused a 202 bp duplication (red line). Arrow tips mark the 3’-end of the genes

By analyzing strain-specific regions in Oxford Nanopore-derived *M. hominis* genome sequences, a hitherto undiscovered RM system was detected in *M. hominis* strain 16753VA. It was homologous to the *Hga*I-RM system of *Haemophilus* *gallinarum* (synonym *Avibacterium volantium*) [41]. BLASTN analysis identified the presence of this RM system in the published genomes of *M. hominis* strains PX1114 (acc.-no. CP032849.1) and MIN-132 (CP086131.1). As schematically shown in Fig. 1, the 4.8 kb *mho*VI cassette of *M. hominis* was positioned in the genomic region between the core genes MHO_3110 and MHO_3120 with respect to type strain PG21 and was composed of the three *hga*I-homologous genes, encoding two methyltransferases (M1.*Mho*VI and M2.*Mho*VI), a restriction endonuclease (R.*Mho*VI) and a 70-amino-acid (AA) XRE family transcriptional regulator gene, positioned upstream of the M1.*Mho*VI gene (Fig. 1).

Multiple sequence alignment of the intergenic regions between MHO_3110 and MHO_3120 in PG21 and PX1114 revealed integration of the *mho*VI-cassette 54 bp upstream of the MHO_3120 3-’end (at position 369,286 of PG21) with duplication of the genomic PG21 region [369,084 to 369,286] upstream [386,670 − 386,982] and downstream [391,808 − 392,002] of the *Mho*VI-cassette of PX1114 (see Fig. 1, red line).

### Conservation of the *mho*VI-RM cassette is high in *M. hominis*

Based on sequence information of the *mho*VI-cassette in *M. hominis* strains 16753VA, PX1114 and MIN-132, qPCR primer pairs were designed for each of the *mho*VI-genes (see Table 1) and subjected to 239 clinical isolates. 12.97% (31/239 isolates) tested positive for each of the four genes (see Supplementary Tab. S4), which is moderate compared to the known 5mC RM systems of *M. hominis* ranging from 2.39% (*Eco*47II-homolog RM.*Mho*V) to 27.2% (*Hha*I-homolog RM.*Mho*IV) [30]. Oxford Nanopore sequencing of the genomes of eight *mho*VI-positive isolates was performed and confirmed the conserved architecture and genomic position of the *mho*VI-genes cassette as shown for strain PX1114 (Fig. 1). To decipher error-free gene sequences of the *mho*VI-cassette region of nine isolates, overlapping PCR products were sequenced according to Sanger [42]. Intra-species conservation of the total *mho*VI-gene cassette was high (99.89% nucleotide identity) and homology of the deduced protein sequences was highest for the MTases (99.93% for M1.*Mho*VI and 99.96% for M2.*Mho*VI), followed by 99.83% for R.*Mho*VI) and 99.65% for XRE (see Table 3, A and B).

**Table 3:**
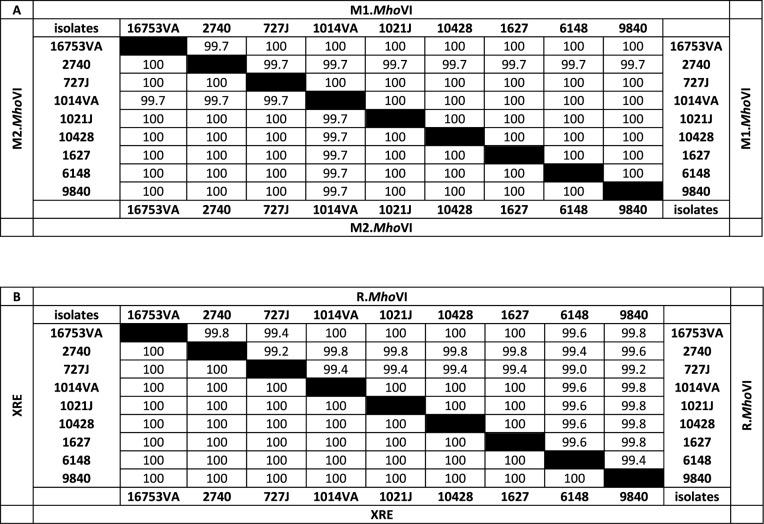
Intra-species homology (%) of the *Mho*VI-RM enzymes

XRE, the smallest protein consisting of 70 amino acids (aa) with a calculated molecular mass of 7.88 kDa, exhibited the highest intra-species conservation with no amino acid substitutions. The highest divergence of the RM genes was observed for the restriction endonuclease R.*Mho*VI, the largest protein with 520 aa and a molecular mass of 60.3 kDa, with a total of 5/520 aa changes. M1.*Mho*VI and M2.*Mho*VI were of similar size with 362 and 358 aa, respectively, and a calculated molecular mass of 41.3 kDa for both. Homology of *M. hominis* M1.*Mho*VI and M2.*Mho*VI was 49% throughout all tested *M. hominis* genomes (Supplementary. Tab. S1). Phylogenetic analysis of the amino acid sequence of RM.*Mho*VI MTases and RM.*Hga*I MTases, including M1 and M2 homologs of *Hga*I in *M. lactucae*, *H. pylori*, *L. petauri*, that comprised the region from the first conserved domain (Motif I) to the last (Motif X), revealed a monophyletic M2 branch (see Supplementary Fig. S3A). In contrast, M1-MTases clustered paraphyletically with M1.*Mho*VI, showing a similar homology to M1.*Hga*I and M2.*Hga*I. Comparable results were obtained when analyzing the region between motif VIII and motif X, which includes the target recognition domain (TRD) responsible for DNA-binding and motif specificity (Fig. S3B) [43]. Due to the perfect clustering of M2-MTases in a common branch, it was assumed that M2.*Mho*VI methylates motif GA^m^CGC and, even though M1.*Hga*I-homologs seem to be more heterogeneous, M1.*Mho*V1 was assigned to the complementary sequence motif G^m^CGTC.

### XRE is only in *M. hominis* part of the *Hga*I-homologous RM cassette

Homologs of M1.*Mho*VI, M2.*Mho*VI, and R.*Mho*VI were also detected in other bacteria: *Mesoplasma lactucae* (acc-.no GCF_002441935.1), a mollicute isolated from the plant *Lactuca sativa*; the human pathogen *Helicobacter pylori* (GCF_002831845.1); the marsupial and fish-colonizing *Lactococcus petauri* (GCF_023499275.1), and the avian *H. gallinarum* which gives the *Hga*I-RM system its name. Thus, neither an evolutionary spread of the *Mho*VI-RM element in a common phylogenetic branch nor a common anatomic site of colonization is obvious. The analysis of inter-species conservation revealed varying degrees of similarity across the different enzymes (Table 4).

**Table 4:** Inter-species homology (%) of the RM.*Mho*VI enzymes

RM.MhoVI-homolog in organism	RM.MhoVI of Mycoplasma hominis PX1114		
	M1	M2	*R*
*Mesoplasma lactucae*	68.5	71.0	49.5
*Helicobacter pylori*	58.0	66.7	52.0
*Lactococcus petauri*	46.5	50.9	26.8
*Haemophilus gallinarum*	46.6	48.1	27.4

**Fig. 2:**
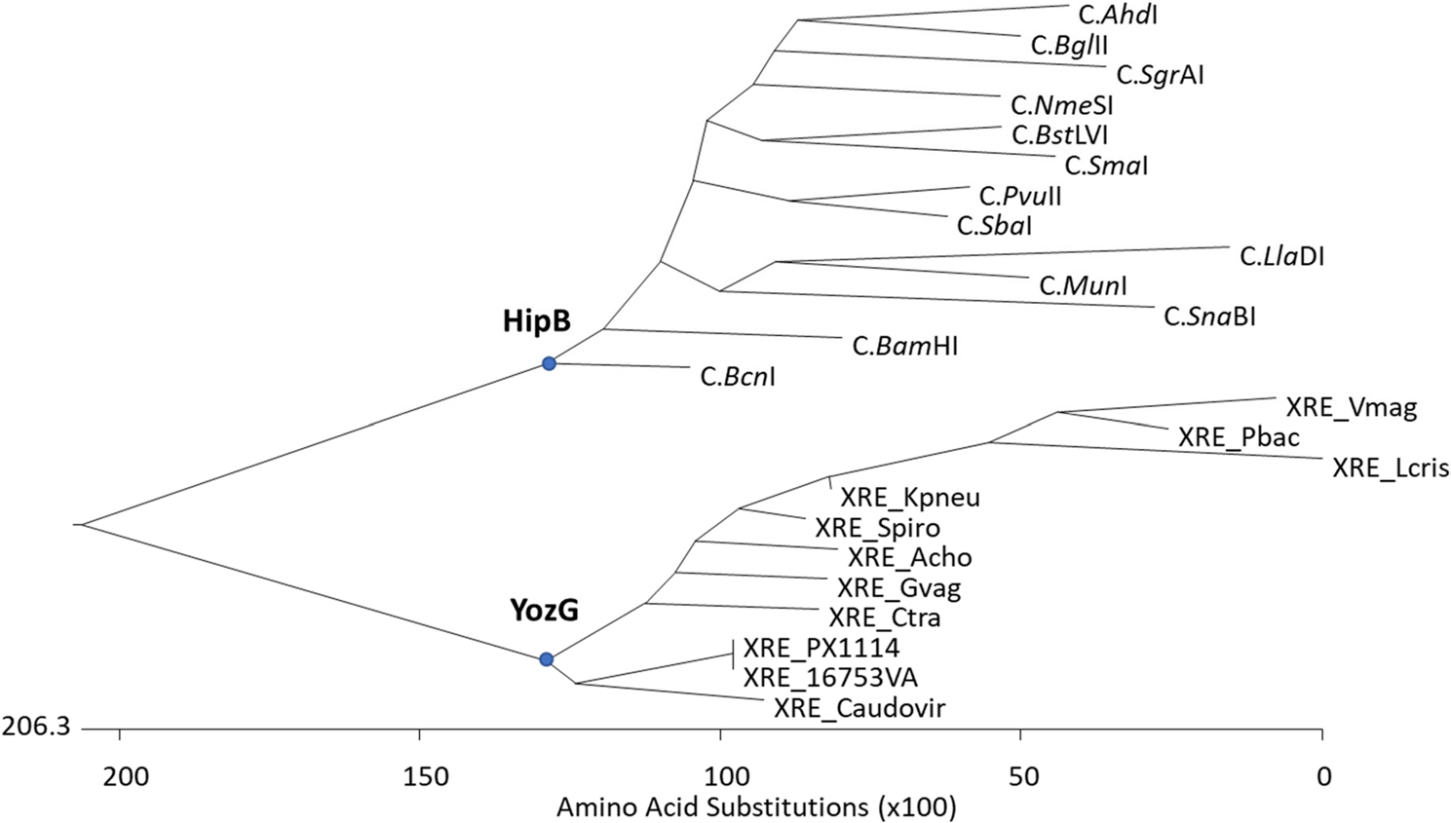
Phylogenetic tree of selected XRE.*Mho*VI-homologs​. The construction of the phylogenetic tree was performed with MegAlign 5.08; accession numbers and abbreviations of the proteins are deposited in Supplementary Table S2

The amino acid conservation of the homologs ranged from 46.5 to 68.5% to M1.*Mho*VI, 48.1–71% to M2.*Mho*VI, and 27.4–49.5% to R.*Mho*VI, demonstrating that the REases were more divergent than MTases. The MTases of *Mycoplasma hominis* strain 16753VA, used as reference, exhibited the highest homology to *Mesoplasma lactucae* (M1.*Mho*VI: 68.5%; M2.*Mho*VI: 71%), followed by *Helicobacter pylori* (M1.*Mho*VI: 58%; M2.*Mho*VI: 66.7%), *Lactococcus petauri* (M1.*Mho*VI: 46.5%; M2.*Mho*VI: 50.9%), and *H. gallinarum* (M1.*Mho*VI: 46.6%; M2.*Mho*VI: 48.1%). Notably, inter-species conservation of M2.*Mho*VI was on average 4.3% higher than of M1.*Mho*VI. Interestingly, XRE.*Hga*I sequence homologs were not found in RM.*Hga*I homologs of other procaryotes, but were associated with other putative RM systems. Analysis of XRE.*Mho*VI homologs revealed their affiliation with members of the *Yoz*G subfamily within the XRE family. Phylogenetic reconstruction further demonstrated that C proteins are closely related to XRE.*Mho*VI but form a distinct monophyletic clade *Hip*B, clearly separated from the *Yoz*G-like XRE subfamily branch (Fig. 2).

The members of the two branches differ not only in sequence homology but also in their genomic organization regarding the relative position of the transcriptional regulator gene in the respective RM cassette. In the *Hip*B-associated branch, the genes encoding controller proteins are located directly upstream of the REase genes, while the corresponding MTase genes are typically found on the opposite DNA strand (see Supplementary Tab. S2). In contrast, members of the *Yoz*G subfamily, such as XRE.*Mho*VI, exhibit a conserved gene arrangement, in which the transcriptional regulator is followed by the MTase gene and then the REase gene.

### *Mho*VI-gene transcript levels support a polycistronic organization

**Fig. 3:**
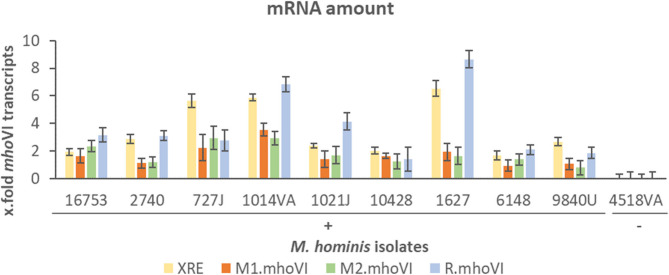
Transcripts of *mho*VI-genes in *M. hominis. *The mRNA levels of the mhoVI-genes were quantified relative to the mean transcript level of gap and lgt using the ΔCT method. Error bars representing standard deviations were calculated on the base of two biological and one technical replicate; each measured in duplicates. Displayed are the results from nine RM.mhoVI positive (+) and one RM.mhoVI negative isolate (-) for comparison

Expression of the *Mho*VI-RM system was investigated by the quantification of transcript amounts relative to the housekeeping genes *gap* and *lgt*; for each *mho*VI-gene using primer pairs binding within each gene. Transcription of the *mho*VI-genes was higher than that of the housekeeping genes in all RM.*Mho*VI positive isolates (Fig. 3).

The expression ranged from 0.78 ± 0.5 (M2.*mho*VI in isolate 9840U) to 8.65 ± 0.62 (R.*mho*VI in isolate 1627). M1.*mho*VI and M2.*mho*VI were the least transcribed on average with 1.72 ± 1.61 and 1.80 ± 1.64, respectively, while R.*mho*VI showed the highest relative expression at 3.78 ± 1.75. The differences in transcript levels of the *mho*VI-genes calculated by one-way ANOVA test (*p*-value > 0.05) showed no overall statistical significance. However, looking at the transcription profile of the four genes in each isolate, the M1.*mho*VI and M2.*mho*VI transcript levels were approximately equal, and generally lower than those of XRE and R.*mho*VI. As expected, no significant transcript levels of RM genes were detected in the nine RM.*Mho*VI negative isolates, as shown for isolate 4518VA as a representative (Fig. 3).

**Fig. 4:**
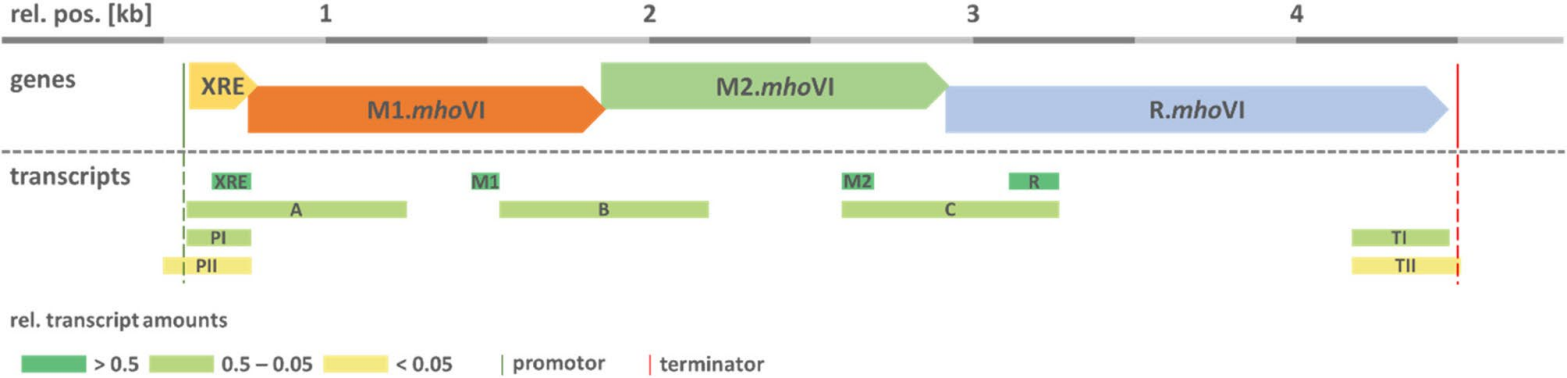
RT-qPCR analysis of gene overlapping *Mho*VI-regions. Schematic representation of the 4.8 kb *Mho*VI gene locus with PCR products numbered according to Table 2. The levels of *Mho*VI-specific gene-, promotor- and terminator-overlapping transcripts were calculated relative to *gap* transcripts and rated to the nearest power of ten (< 0.05, 0.05–0.5, > 0.5). Bioinformatically predicted promoter (green) and terminator (red) positions are indicated by dashed lines. All positions (rel. pos.; 1 bp − 4.825 kb) correspond to the *mho*VI-gene-cassette region from nt 391,807 – nt 386,982 in PX1114 (CP032849.1)

To test the hypothesis of a polycistronic mRNA, gene-overlapping RT-qPCRs were conducted using primers that were designed to produce PCR products that extend at least 300 nt across two adjacent genes (Fig. 4).

Gene-overspanning RT-qPCR products (A-C) were quantified using the ΔCT method. Because amplicon efficiency is affected by primer pair characteristics and the length of RT-PCR products, simultaneous amplification of genomic DNA was performed to normalize transcript levels. Amplicon (A) started 8 bp upstream of XRE, spanned the XRE gene completely and covered 474 nt of the M1.*mho*VI gene. Amplicon (B) spanned 329 nt M1.*mho*VI 3’-end and 323 nt M2.*mho*VI 5’end; and amplicon (C) covered 333 nt M2.*mho*VI (3’-end) and 374 nt R.*mho*VI (5’-end). Overlapping amplicons (A, B, C) were detected at the three gene junctions, supporting the thesis of a polycistronic *mho*VI-cassette mRNA.

Two potential universal promoter regions were identified upstream of the XRE gene. The first was detected by Sapphire at the relative nucleotide position 531–559 with a *p*-value of 0.0002, the second by ProPr2.0 in an overlapping region at nt 496–567 with the highest possible score of 1. In RT-qPCR analysis, a 200 bp product was generated, starting upstream of the suspected promoter at position nt 571 (Fig. 4; PI), whereas a 274 bp product, starting downstream at position nt 491 (Fig. 4; PII), was barely detected (ΔCTs of 0.22 and 0.007, respectively), strongly supporting the transcription promoter site between position 497 and 571.

Using the browser tool ARNnold, a universal transcription terminator was detected downstream of *R.mho*VI at nt 4470–4519, with a moderate ΔG-score of −7.3. qPCR analysis supported the assumption of a terminator region downstream of nt 4473, as a 302 bp PCR product (Fig. 4; TI) extending to nt 4473 was synthesized in significant amounts (ΔCT of 0.126), while a 337 bp product (Fig. 4; TII) extending to nt 4508 was synthesized in significantly lower amounts (ΔCT of 0.026; Fig. 4).

### RM.*Mho*VI positive isolates always carry GA^m^CGC/G^m^CGTC-methylated DNA

**Fig. 5:**
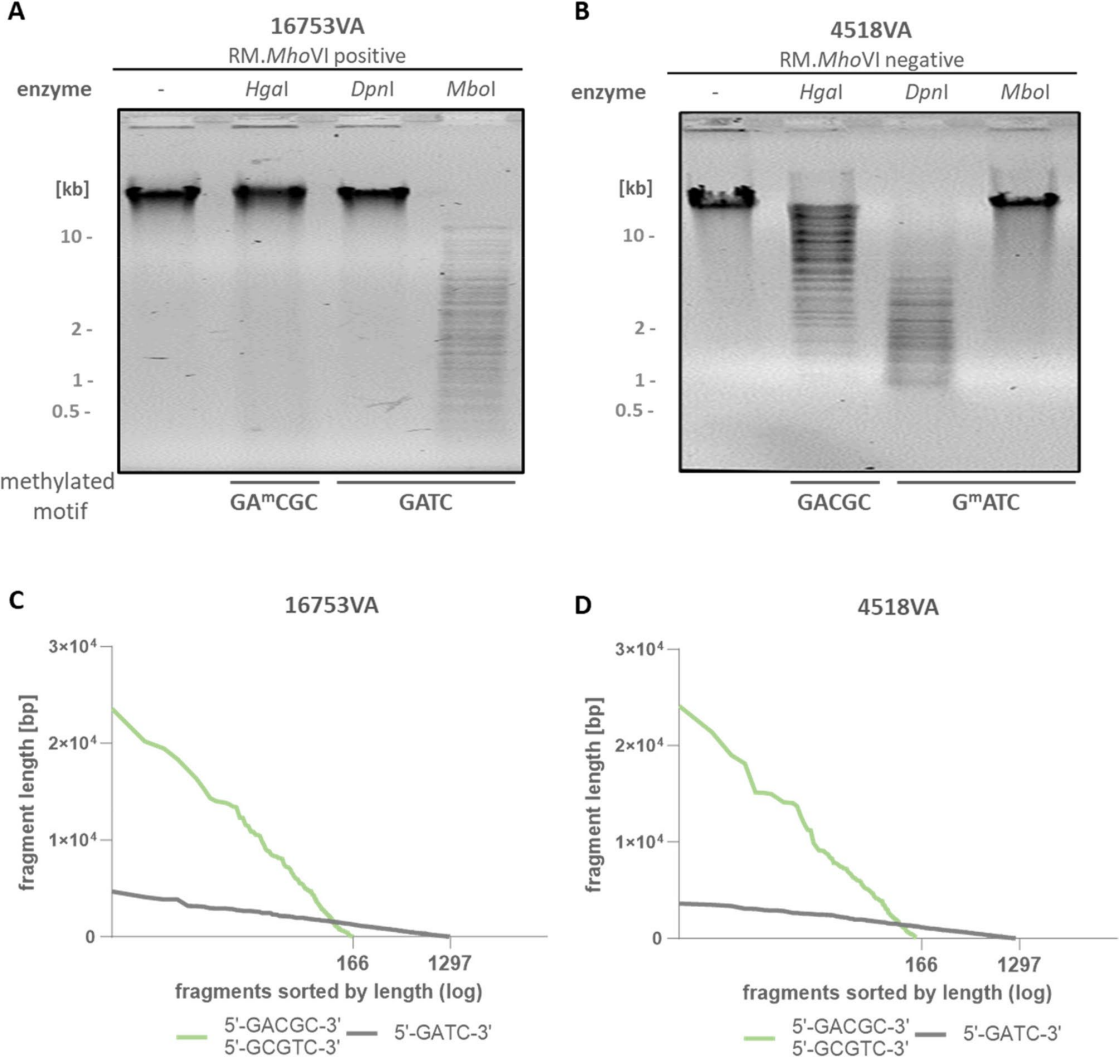
Methylation-sensitive restriction analysis. DNA of a RM.*Mho*VI positive (**A**, 16753VA) and a negative strain (**B**, 4518VA) was restricted by methylation-sensitive restriction (MSR) enzymes *Hga*I (GACGC/GCGTC), *Dpn*I (G^m^ATC) and *Mbo*I (GATC) and separated on agarose gels. ^m^ = methylated base **(C - D**) Simulation of total DNA restriction by *Hga*I (green line) or *Dpn*I/*Mbo*I (grey line) of isolates 16753VA **(C) **and 4518VA **(D)**. The number of fragments is plotted against fragment length

To demonstrate the activity of the native *Mho*VI-MTases of *M. hominis*, the methylation state of the genomic DNA of *Mho*VI-RM positive and negative isolates was investigated in a methylation-sensitive restriction (MSR) analysis. The restriction enzyme *Hga*I, which restricts only unmethylated GACGC/GCGTC motifs was utilized to determine 5mC methylations within the presumed RM.*Mho*VI recognition motif (Fig. 5).

As shown in Fig. 5A, lack of DNA restriction by the endonuclease *Hga*I was observed in the *Mho*VI-RM-positive isolate 16753VA, indicating protection of the DNA by 5mC methylation of the *Mho*VI-recognition motif GACGC/GCGTC and thus suggesting methylation activity of the native *Mho*VI-MTases. Restriction of the DNA of strain 4518VA by *Hga*I demonstrated a lack of 5mC methylation in the GACGC/GCGTC motif, which was consistent with the absence of the *Mho*VI-RM system (Fig. 5B). The restriction patterns of isolates 16753VA and 4518VA corresponded to those of the eight *Mho*VI-positive and -negative tested isolates, respectively (see supplementary Fig. S1 and supplementary Tab. S3). To confirm that the DNA is susceptible to nucleolytic digestion at all, the two restriction enzymes *Mbo*I and *Dpn*I were used, as they restrict the GATC motif in 6 mA-methylated or -unmethylated form, respectively.

**Fig. 6:**
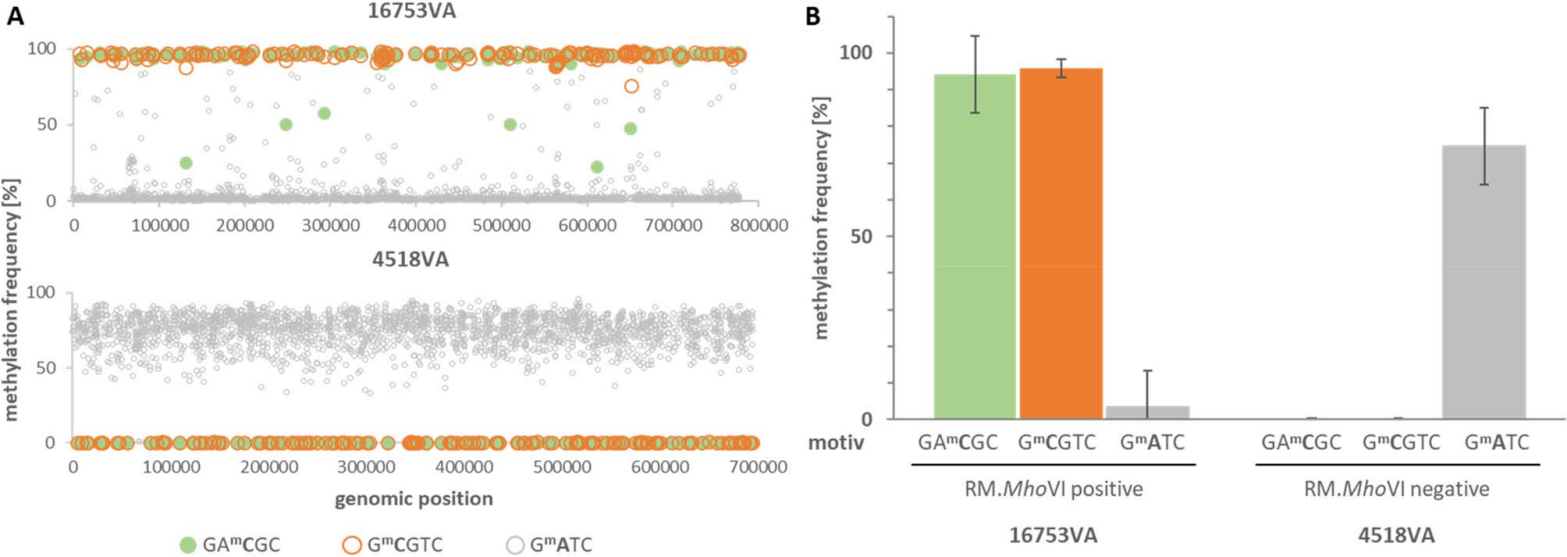
Nanopore-derived methylation frequencies of GA^m^CGC, G^m^CGTC and G^m^ATC. **(A)** Dot plots show the percentage of 5mC modified bases (methylation frequency) in the RM.*Mho*VI recognition motifs GACGC (green) and GCGTC (orange) as well as 6 mA methylation frequency of GATC (gray) at their respective genomic positions in the RM.*Mho*VI-positive isolate 16753VA and the RM.*Mho*VI-negative isolate 4518VA. **(B)** Bar graphs show the average methylation frequency of all GA^m^CGC (green), G^m^CGTC (orange) and G^m^ATC (gray) which were identified in Dorado analysis of the Nanopore sequenced genomes of isolate 16753VA and 4518VA.

The GATC-specific restrictions seemed to fragment the DNA more extensively compared to *Hga*I. This could be attributed to a reduced number of GC-enriched motifs (such as GACGC) in the genome (Fig. 5C-D), which corresponds to the reduced GC content of mycoplasma genomes ranging from 21 to 40% [44]. As expected, the calculation of total DNA restriction produced approximately eight-fold more GATC-fragments with mean lengths of 600 bp than GACGC-fragments of meanly 4600 bp. Thus, it was hypothesized that the MTases of *Mho*VI methylated *each* recognition motif in the genome of *Mho*VI-positives. To prove this hypothesis, the Oxford Nanopore reads of the sequenced genomes of strains 16753VA and 4581VA were analyzed to calculate 5mC and 6 mA modifications across the genomes. As M1.*Hga*I of *H. gallinarum* is known to methylate G^m^CGTC and M2.*Hga*I the complementary GA^m^CGC motif, the methylation frequencies (MF) of both motifs were analyzed independently and that of G^m^ATC as a control (Fig. 6) [26].

As shown in Fig. 6A, high methylation rates (> 95%) of both, 5’-G^m^CGTC-3’ and 5’-GA^m^CGC-3’, were observed at almost every motif site in the RM.*Mho*VI positive isolate 16753VA, but below background in the RM.*Mho*VI free isolate 4518VA. In contrast, 5’-G^m^ATC-3’ methylation was below background in isolate 16753VA, but positive in isolate 4518VA, which had recently been shown to express (in contrast to 16753 A) a G^m^ATC methylation by Dam1 (Fig. 6B) [30]. Interestingly, six positions were identified in isolate 16753VA with an apparent hemimethylation, as the methylation rate of GA^m^CGC were lower than that of G^m^CGTC-by up to 70% (Fig. 6A, green dots).

### Methylation at GA^m^CGC is generally higher than at G^m^CGTC.

**Fig. 7:**
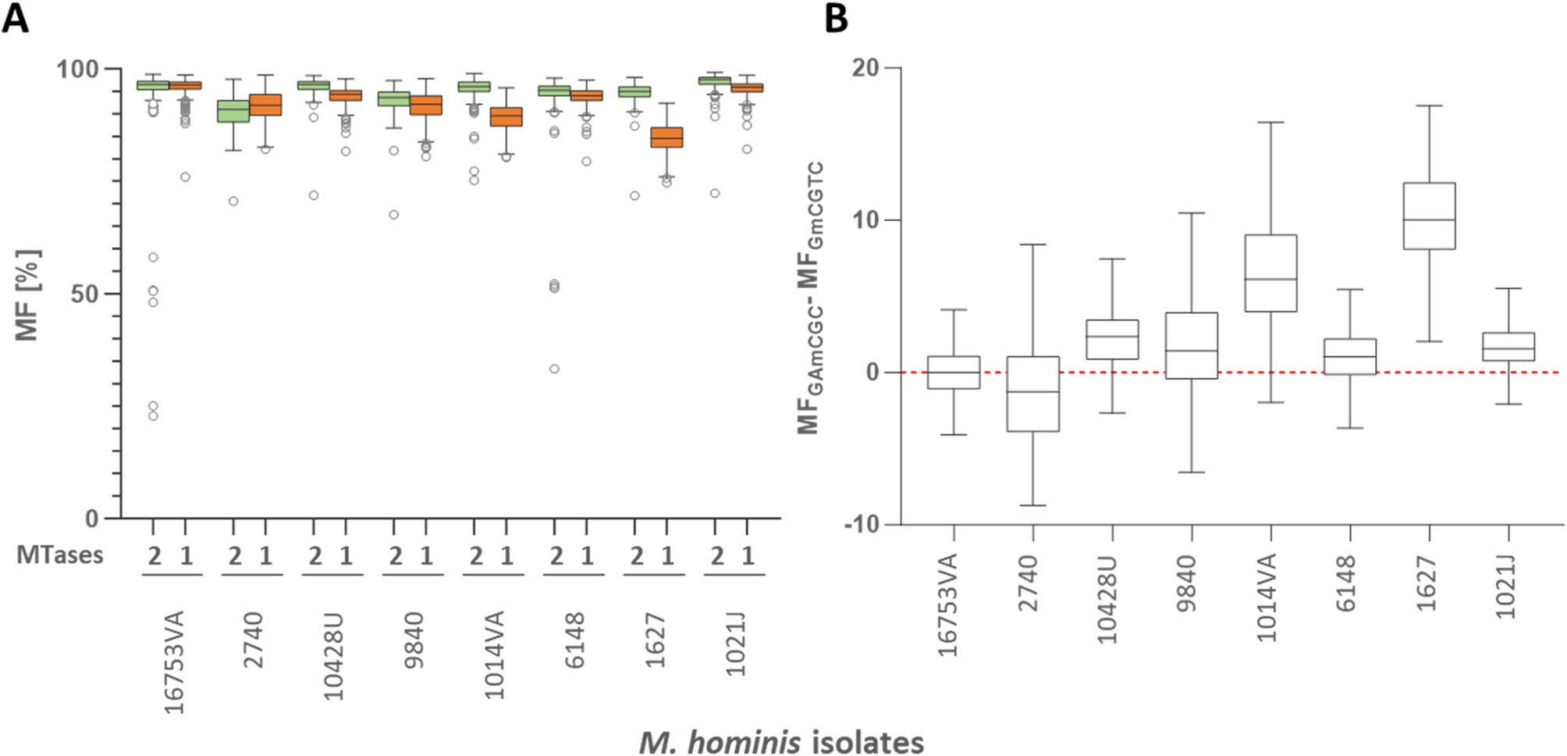
Strain-specific GA^m^CGC [2] and G^m^CGTC [1] methylation frequencies. **(A)** Tukey box plots showing the proportion of methylated motifs (methylation frequency [% MF]) of GA^m^CGC (2, green) and G^m^CGTC (1, orange) for each of eight *Mho*VI-positive isolates. **(B)** Boxplots of the average MF differences between M2.*Mho*VI and M1.*Mho*VI at each motif site of the isolate‘s genome sequence. Wilcoxon signed-rank test calculated high significance (*p* < 0.0001) for all isolate but 16753VA (*p* > 0.05)

Average methylation rates of G^m^ACGC, G^m^CGTC and G^m^ATC were then calculated for each *Mho*VI-positive isolate (Fig. 7A and Supplementary Tab. S3). A putative hemimethylation of the non-palindromic RM.*Mho*VI-motif, which was observed for six out of 166 motifs in isolate 16753VA, was not calculated for other isolates; However, analysis of eight *Mho*VI-positive isolates revealed differences in the average methylation rates of GA^m^CGC and G^m^CGTC in all but the reference isolate 16753VA (Fig. 7A).

With respect to the differences in methylation rates of GA^m^CGC or G^m^CGTC at each motif site, Wilcoxon signed-rank test was performed to verify statistical significance (Fig. 7B). Methylation discrepancies (MF_GAmCGC_-MF_GmCGCT’_) were calculated in seven out of eight isolates were highly significant (*p*-values < 0.0001). A non-significant difference of 0.01 (*p* > 0.05) was only detected in isolate 16753VA. In six of eight isolates, GA^m^CGC methylation exceeded G^m^CGTC methylation ranging from 1.05 in isolate 6148 to 10.03 in isolate 1627. Isolate 2740 exhibited a reverse pattern exhibiting higher G^m^CGTC than GA^m^CGC activity, suggesting isolate-specific factors affecting MTases’ activity.

### Recombinant M2.*Mho*VI exhibits G^m^ACGC methylation activity

**Fig. 8:**
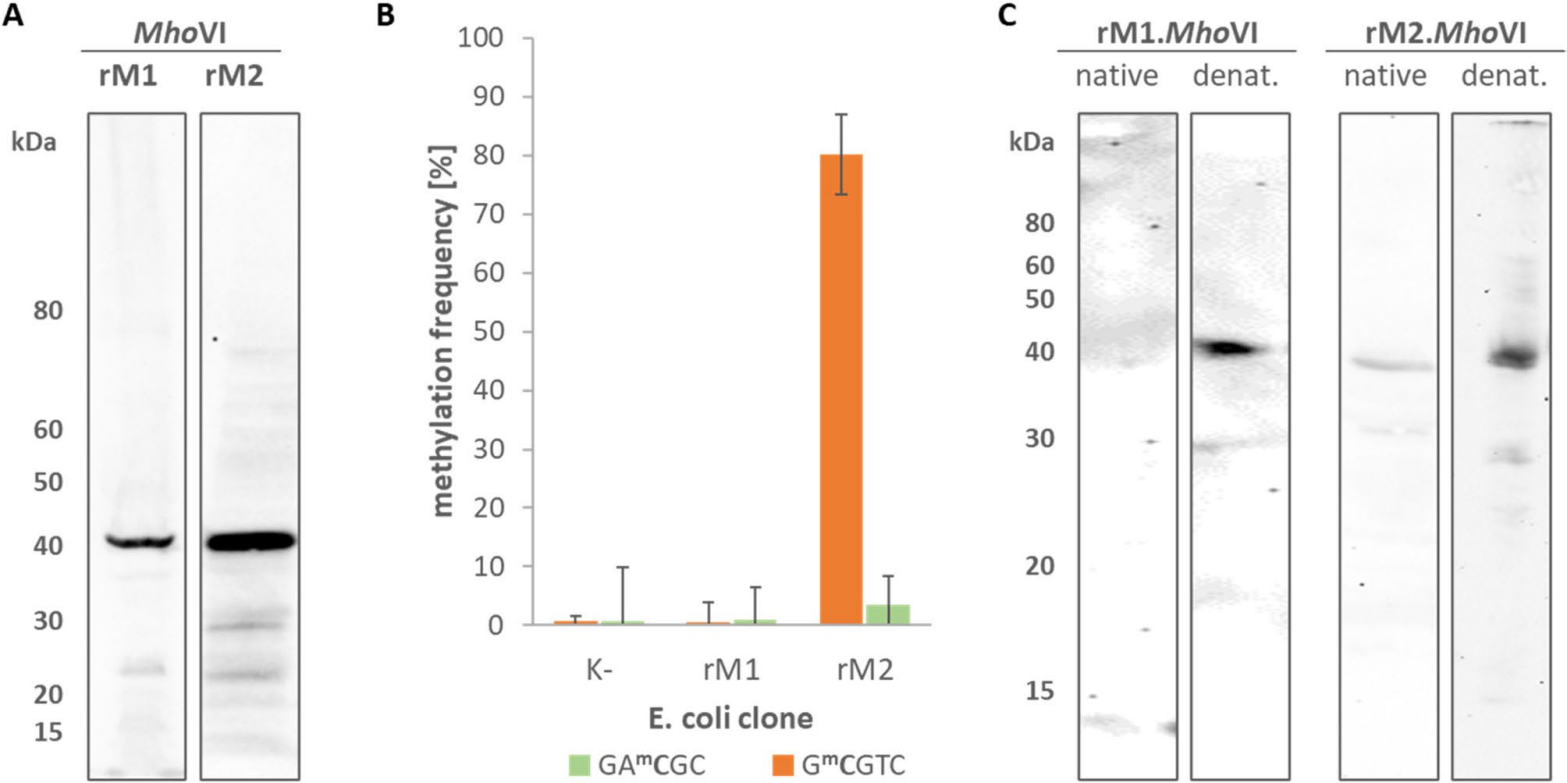
Analysis of recombinant *Mho*VI-MTases. **(A)** Protein lysates of *E. coli* clones, expressing recombinant rM1.*Mho*VI and rM2.*Mho*VI, were separated on 4–12% gradient SDS-PAGE gels, blotted and visualized via immunostaining with His_6_-antibody. **(B)** Mean methylation frequencies of GA^m^CGC and G^m^CGTC motifs in DNA of these *E. coli* clones and of an untransformed *E. coli* (K-) as a control, **(C)** *E. coli* cells expressing rM1.MhoVI or rM2.*Mho*VI were lysed under native or denaturating (denat.) conditions. The fraction of soluble proteins separated on 12% SDS-PAGE followed by western blotting and His₆-antibody staining

To confirm the methylation activity and motif specificity of the RM.*Mho*VI MTases as *Hga*I-MTases homologs, the two MTases were cloned into expression vectors and propagated into *E. coli*. Sanger sequencing confirmed the presence and open reading frame of the inserts, but revealed mutations that led to amino acid changes. The recombinant rM1.*Mho*VI carried the one isofunctional substitution and (D304E), as well as two non-isofunctional substitution (R195G and R268G). Both proteins were heterologous expressed in *E. coli* by IPTG induction and detected in immunoblotting, with rM2 showing a higher expression level than rM1 (Fig. 8A).

Subsequent nanopore sequencing on the genomic DNA of these *E. coli* clones demonstrated significant GA^m^CGC methylation of the rM2 clone DNA (MF 80.1%; Fig. 8B), and a minimal G^m^CGTC methylation (MF = 3.4%). Both motifs, GACGC and GCGTC, were found to be unmethylated in the rM1 clone DNA (MF < 1%). As shown in Fig. 8C, both recombinant proteins were mainly soluble only under denaturing conditions. However, small amounts of rM2, which showed higher expression, were also detected in the soluble fraction following native lysis. This suggests that both rMTases are predominantly expressed in (methylation-inactive) inclusion bodies, with limited solubility of (presumably active) rM2 under native conditions.

## Discussion

Sequence homology, conserved genomic organization of the *Mho*VI-RM system, and the detection of methylated DNA motifs in *M. hominis* consistent with those of the *Hga*I RM system in *H. gallinarum* collectively suggest that RM.*Mho*VI represents the *Mycoplasma hominis* homolog of RM.*Hga*I. At last, functional analysis of the recombinant methyltransferase rM2.*Mho*VI proved its target motif as GCGTC, which is identical to that of M2.*Hga*I, thereby confirming the proposed orthology.

In most type II RM systems, the common recognition motif of REase and MTase is palindromic and enables methylation on both DNA strands by a single MTase. However, there are examples, like the *Hha*I-homolog RM.*Mho*IV, that (like *Hga*I) harbors two 5mC MTases, but recognizes a palindromic sequence motif GCGC [30, 45]. RM systems like the *Hga*I RM system belong to the subclass of Type IIS RM systems, which is characterized by (1) recognition of a non-palindromic motif, mostly methylated by two individually acting MTases and (2) restriction of the DNA outside the recognition sequence by the endonuclease [45, 46]. As RM.*Hga*I homolog, RM.*Mho*VI of *M. hominis* was therefore assigned as Type IIS.

Analysis of heterologous expressed proteins rM1 and rM2 in *E. coli*, confirmed that the target motif of rM2.*Mho*VI is identical to that of M2.*Hga*I, GCGTC. No methylation activity of rM1.*Mho*VI could be detected under the tested conditions. Notably, the low methylation frequency of GACGC (3.4%) observed in the rM2 clone suggests that this motif is not the primary target of rM2.MhoVI, but rather reflects a weak off-target activity, as the frequency slightly exceeded the background signal (~0.7%). Similar off-target methylation events have been reported for the GATC-specific 6 mA MTases M.*Eco*KDam and MAGa2700 methylating GACC and AATC or YRATC and RGATC (Y = C or T; R = A or G), respectively [47, 48]. The results of this study demonstrate that rM2.*Mho*VI functions independently of rM1.*Mho*VI and suggest that M1.*Mho*VI is responsible for GACGC methylation in *M. hominis*, consistent to the function of M1.*Hga*I.

The absence of detectable rM1 activity may be unlikely attributed to the detected amino acid mutations, as secondary structure predictions did not indicate a misfolded protein (data not shown). Furthermore, the mutations were not located within known functional domains of Type II-DNA MTases (see supplementary Fig. S3A) [49]. It is more likely the result of a weak expression, poor solubility and aggregation into inclusion bodies in *E. coli*. In contrast, M2.MhoVI displayed methylation activity despite also forming inclusion bodies. This is likely due to its higher expression level leading to a significant proportion of soluble, functional protein. Contrarily to the traditional view that inclusion bodies consist solely of misfolded, inactive proteins, recent studies have shown that in some cases they can contain substantial amounts of correctly folded, active proteins [50, 51]. Moreover, the proportion of active protein within inclusion bodies can vary depending on factors such as cultivation temperature and bacterial growth phase [52, 53]. In accordance with this, but in contrast to the *Mho*VI-RM system, we found methylation activity of recombinant MTases of another RM system, despite their expression in inclusion bodies (manuscript in preparation).

Differences in expression levels observed for the recombinant proteins rM1.*Mho*VI and rM2.*Mho*VI in *E. coli* may reflect differential expression and activity in the native host. Since the MF of GA^m^CGC was higher than the MF of G^m^CGTC in six out of eight *M. hominis* isolates it can also be hypothesized that on average M2.*Mho*VI exhibits higher methylation activity than M1.*Mho*VI in their native environment. Analyses on protein level will help to elucidate the basis of MTase activity differences in future work.

Interestingly, the number of MTases in homologous RM systems can vary from species to species: in contrast to *Mho*V- and *Mho*VI-, the *Hha*I-RM system of *H. haemolyticus* consists of just one MTase [54], and the *Hga*I-RM system, *Nme*BI, of *N. meningitis* still harbors a single M1.*Hga*I-homolog MTase [55], respectively. A hemimethylation of the DNA was shown to be sufficient to maintain defense of the RM system: the data of Sugisaki et *al*. showed that methylation of just GA^m^CGC or G^m^CGTC retains protection against the REase [26], and a change of the specificity of the *Hha*I-MTase from GCGC to GCG revealed full protection of the DNA by the biotechnologically designed hemimethylase [56]. However, methylation of both strands is of utmost importance in DNA replication. In the case of hemimethylation, synthesis of the strand carrying the unmethylated motif leads to a completely unmethylated recognition motif downstream of the replication fork, which is then susceptible to double-strand breaks by the REase [8]. Therefore, a regulatory mechanism for balanced MTase and REase expression is beneficial to prevent DNA degradation, not only in the case of replication.

The regulation of RM systems is complex and can occur at both the transcriptional and translational level [11–14]. XRE may be a species-specific regulator for *Hga*I.RM expression. Average transcript levels of XRE and R.*mho*VI exceeded those of M1.*mho*VI and M2.*mho*VI, suggesting that additional internal promoter and terminator sites might actively influence transcription. This points to a mixed transcriptional organization, with potentially monocistronic expression of XRE.*mho*VI and R.*mho*VI embedded within an otherwise polycistronic RM locus (see Supplementary Table S4), a structure also described for RM.*Eco*29kI [12].

Alternatively, post-transcriptional processes such as RNA degradation may account for the observed differences in transcript abundance. Similar regulatory mechanisms have been proposed for RM.*Mho*II and demonstrated for RM.*Eco*P1I and RM.*Eco*P15I, where REase expression is modulated in response to intracellular MTase levels [30, 57].

Transcription factors like the XRE elements are widespread among prokaryotes, where they are involved in stress response and virulence by either enhancing or inhibiting the transcription of downstream genes, e.g. in *Photorhabdus luminescens* and *Streptococcus* sp [58–64]. They are also involved in the regulation of toxin-antitoxin (TA) systems in *Brucella abortus* by repressing the zinc-dependent metalloproteinase (ZnMP) toxin under normal conditions [65]. As RM systems function similar to TA systems, where restriction endonucleases act as toxins and MTases as antitoxins, XRE is also known to interact with the transcription of nuclease genes [66]. In *Caulobacter*, the RM system associated proteins CCNA_00744 and CCNA_01405, exhibit an 11- and 2-fold increase in XRE-deletion strains [67]. In the *Mycoplasma gallisepticum* Mgas6I RM system, the controller protein HsdC has been shown to contain an XRE-like domain and to bind promoter regions within the RM operon to modulate the expression of RM genes [68]. This serves as an example of RM system regulation by members of the XRE family in *mycoplasma* species. In *M. hominis*, the role of XRE as a potential regulator of RM.*Mho*VI remains unclear, but genomic features may offer insights into its regulatory function.XRE family members of the *Hip*B branch are positioned directly upstream of the REase gene to regulate its expression, while the MTase gene is typically located on the opposite strand, suggesting separate regulation [18, 19]. In contrast, *Yoz*G branch members, including XRE.*Mho*VI, show a gene order of XRE-MTase-REase on the same strand. Although the latter group is barely characterized, the different arrangement implies a regulatory mechanism distinct from the *Hip*B group [19]. To investigate the transcriptional regulation of the XRE element, a bacterial one-hybrid system could be used to determine whether XRE interacts with promotor regions of the RM system to regulate REase or MTase expression [62].

In this study, the clinical *M. hominis* strains originated all from arginine cultures in the mid- to late-logarithmic growth phase. Although it has been described that the RM systems are active at this stage, other factors may play a more important role in RM expression that has not yet been analyzed [30]. Further research should also examine RM expression under conditions that mimic the in vivo environment, such as in polymicrobial communities, variations of temperature and pH value or exposure to foreign DNA. Such studies could provide deeper insight into the postulated XRE-based regulation and function of RM.*Mho*VI in pathophysiology and defense mechanism of *M. hominis*.

## Conclusions

A combination of modern ONT whole genome sequencing, bioinformatics and experimental molecular biology was utilized to identify RM.*Mho*VI as the *M. hominis* RM.*Hga*I homolog. The genomic arrangement and MTases activity in the native host resembled those of RM.*Hga*I and functional analysis of recombinant rM2.*Mho*VI confirmed its methylation activity. The detection of a *M. hominis*-specific XRE transcriptional regulator gene within the *Mho*VI-RM cassette suggests a regulatory mechanism in *M.hominis* that is absent in other *Hga*I homologous RM systems.

## Supplementary Information

Supplementary Material 1.

Supplementary Material 2.

## Data Availability

Genome sequences of M. hominis strains PX1114 (acc.-no. CP032849.1) and MIN-132 (CP086131.1) as well as the genomes of Mesoplasma lactucae (acc-.no GCF_002441935.1), Helicobacter pylori (acc-.no GCF_002831845.1) and Lactococcus petauri (acc-.no GCF_023499275.1) were downloaded from NCBI (https://www.ncbi.nlm.nih.gov/nuccore/). Normalized genome sequences from Nanopore assemblies of genomes of clinical *M. hominis* strains are available at OSF (10.17605/OSF.IO/3N4SY). All other data, including Nanopore raw data will be provided on request.
