# Supplementary material for: From sequence to activity: the HgaI-homologous restriction modification system RM.MhoVI of Mycoplasma hominis
Source: BMC Microbiol. 2025 Aug 25;25:543. doi: 10.1186/s12866-025-04270-3 (PMC12376442; doi:10.1186/s12866-025-04270-3)
Supplement: Supplementary file 2 — Supplementary Material 2. [file 12866_2025_4270_MOESM2_ESM.pptx]

## Slide 1
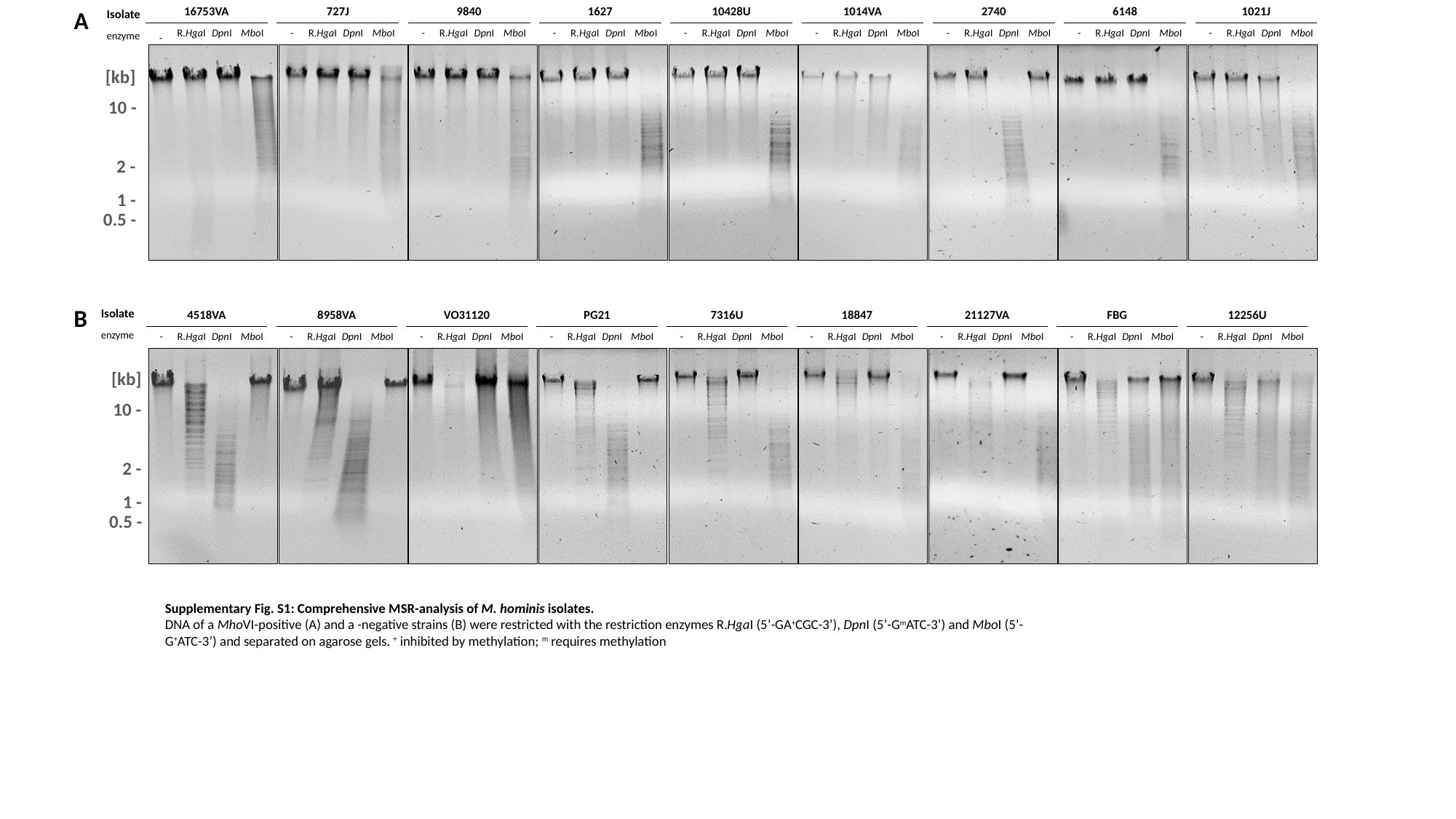

A
| 16753VA | | | | | 727J | | | | | 9840 | | | | | 1627 | | | | | 10428U | | | | | 1014VA | | | | | 2740 | | | | | 6148 | | | | | 1021J | | | |
| --- | --- | --- | --- | --- | --- | --- | --- | --- | --- | --- | --- | --- | --- | --- | --- | --- | --- | --- | --- | --- | --- | --- | --- | --- | --- | --- | --- | --- | --- | --- | --- | --- | --- | --- | --- | --- | --- | --- | --- | --- | --- | --- | --- |
| - | R.HgaI | DpnI | MboI | | - | R.HgaI | DpnI | MboI | | - | R.HgaI | DpnI | MboI | | - | R.HgaI | DpnI | MboI | | - | R.HgaI | DpnI | MboI | | - | R.HgaI | DpnI | MboI | | - | R.HgaI | DpnI | MboI | | - | R.HgaI | DpnI | MboI | | - | R.HgaI | DpnI | MboI |
| Isolate |
| --- |
| enzyme |
[kb]
10 -
2 -
1 -
0.5 -
B
| Isolate |
| --- |
| enzyme |
| 4518VA | | | | | 8958VA | | | | | VO31120 | | | | | PG21 | | | | | 7316U | | | | | 18847 | | | | | 21127VA | | | | | FBG | | | | | 12256U | | | |
| --- | --- | --- | --- | --- | --- | --- | --- | --- | --- | --- | --- | --- | --- | --- | --- | --- | --- | --- | --- | --- | --- | --- | --- | --- | --- | --- | --- | --- | --- | --- | --- | --- | --- | --- | --- | --- | --- | --- | --- | --- | --- | --- | --- |
| - | R.HgaI | DpnI | MboI | | - | R.HgaI | DpnI | MboI | | - | R.HgaI | DpnI | MboI | | - | R.HgaI | DpnI | MboI | | - | R.HgaI | DpnI | MboI | | - | R.HgaI | DpnI | MboI | | - | R.HgaI | DpnI | MboI | | - | R.HgaI | DpnI | MboI | | - | R.HgaI | DpnI | MboI |
[kb]
10 -
2 -
1 -
0.5 -
Supplementary Fig. S1: Comprehensive MSR-analysis of M. hominis isolates.
DNA of a MhoVI-positive (A) and a -negative strains (B) were restricted with the restriction enzymes R.HgaI (5’-GA+CGC-3’), DpnI (5’-GmATC-3’) and MboI (5’-G+ATC-3’) and separated on agarose gels. + inhibited by methylation; m requires methylation

## Slide 2
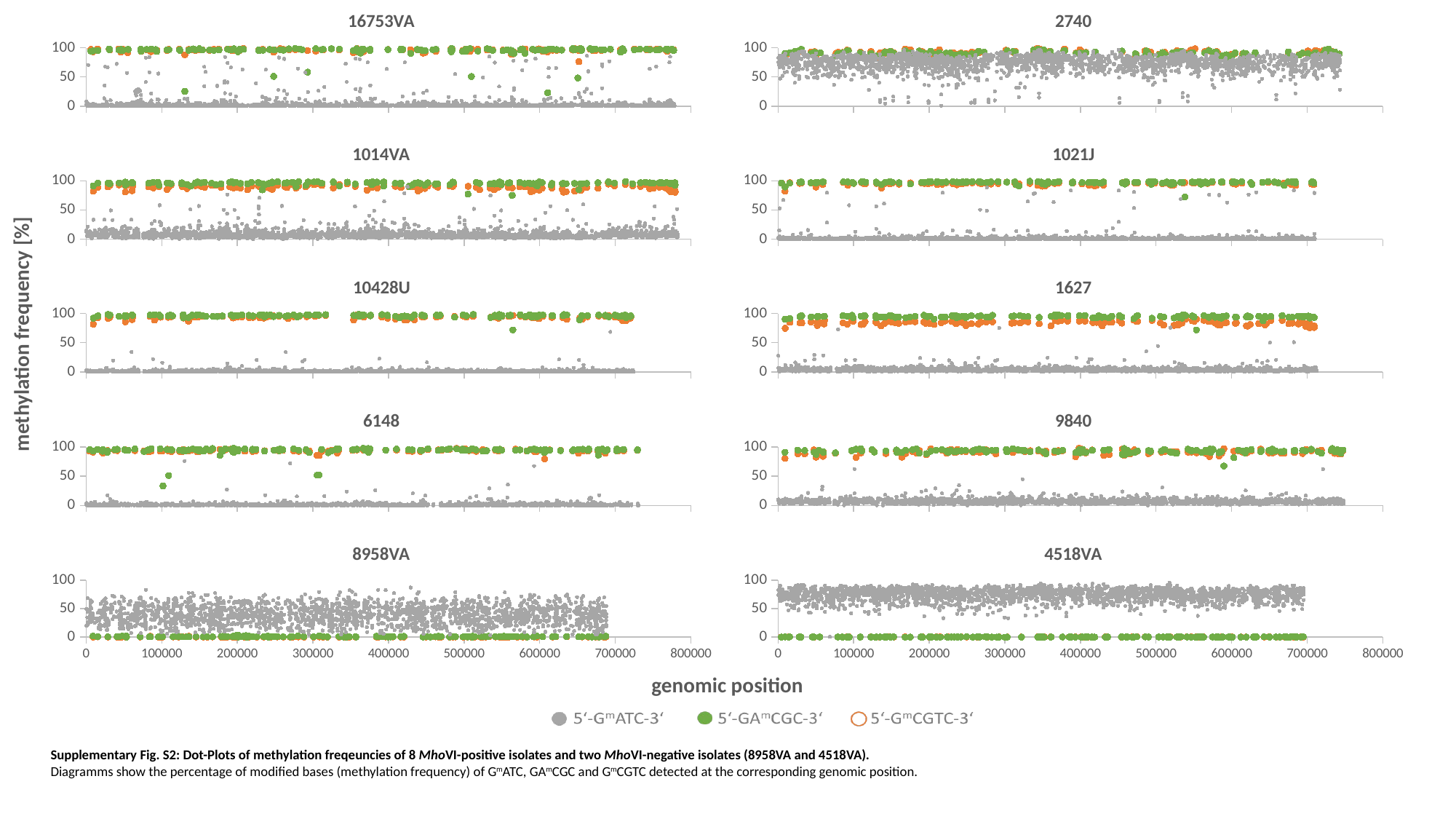

### Chart: 16753VA
| Category | 5'-GmATC-3' | 5'-GAmCGC-3' | 5'-GmCGTC-3' |
|---|---|---|---|
### Chart: 2740
| Category | freq gAtc | freq gaCgc | freq ctgCg |
|---|---|---|---|
### Chart: 1014VA
| Category | freq gAtc | freq gaCgc | freq ctgCg |
|---|---|---|---|
### Chart: 1021J
| Category | freq gAtc | freq gaCgc | freq ctgCg |
|---|---|---|---|
### Chart: 10428U
| Category | freq gAtc | freq gaCgc | freq ctgCg |
|---|---|---|---|
### Chart: 1627
| Category | freq gAtc | freq gaCgc | freq ctgCg |
|---|---|---|---|methylation frequency [%]
### Chart: 6148
| Category | freq gAtc | freq gaCgc | freq ctgCg |
|---|---|---|---|
### Chart: 9840
| Category | 5'-GmATC-3' | freq gaCgc | freq ctgCg |
|---|---|---|---|
### Chart: 8958VA
| Category | freq gAtc | freq gaCgc | freq ctgCg |
|---|---|---|---|
### Chart: 4518VA
| Category | 5'-GmATC-3' | 5'-GAmCGC-3' | 5'-GmCGTC-3' |
|---|---|---|---|genomic position
Supplementary Fig. S2: Dot-Plots of methylation freqeuncies of 8 MhoVI-positive isolates and two MhoVI-negative isolates (8958VA and 4518VA).
Diagramms show the percentage of modified bases (methylation frequency) of GmATC, GAmCGC and GmCGTC detected at the corresponding genomic position.

## Slide 3
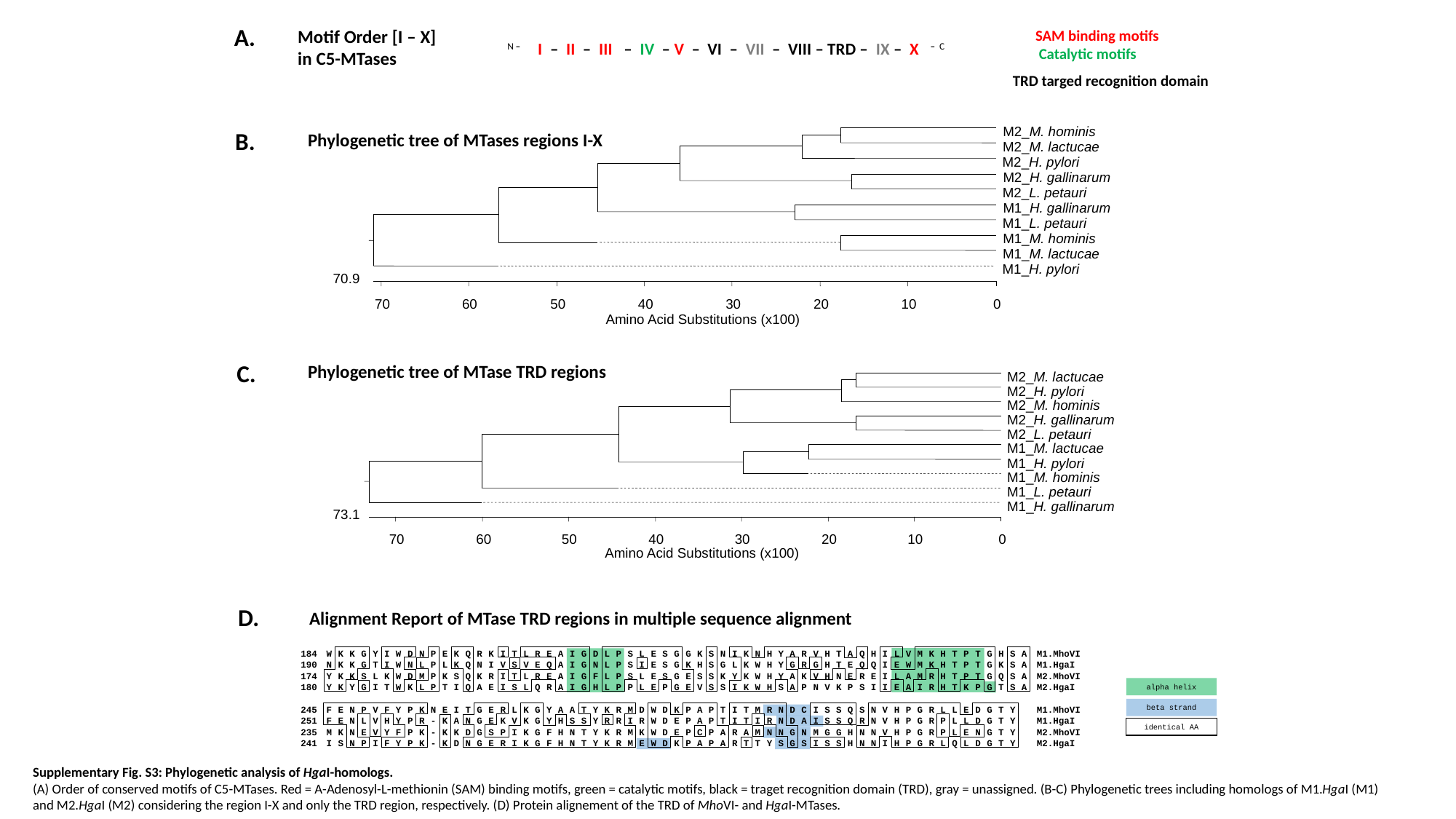

A.
SAM binding motifs
Motif Order [I – X]
in C5-MTases
 N – I – II – III – IV – V – VI – VII – VIII – TRD – IX – X – C
Catalytic motifs
TRD targed recognition domain
B.
M2_M. hominis
M2_M. lactucae
M2_H. pylori
M2_H. gallinarum
M2_L. petauri
M1_H. gallinarum
M1_L. petauri
M1_M. hominis
M1_M. lactucae
M1_H. pylori
70.9
70
60
50
40
30
20
10
0
Amino Acid Substitutions (x100)
Phylogenetic tree of MTases regions I-X
C.
Phylogenetic tree of MTase TRD regions
M2_M. lactucae
M2_H. pylori
M2_M. hominis
M2_H. gallinarum
M2_L. petauri
M1_M. lactucae
M1_H. pylori
M1_M. hominis
M1_L. petauri
M1_H. gallinarum
73.1
70
60
50
40
30
20
10
0
Amino Acid Substitutions (x100)
D.
Alignment Report of MTase TRD regions in multiple sequence alignment
184
W
K
K
G
Y
I
W
D
N
P
E
K
Q
R
K
I
T
L
R
E
A
I
G
D
L
P
S
L
E
S
G
G
K
S
N
I
K
N
H
Y
A
R
V
H
T
A
Q
H
I
L
V
M
K
H
T
P
T
G
H
S
A
M1.MhoVI
190
N
K
K
G
T
I
W
N
L
P
L
K
Q
N
I
V
S
V
E
Q
A
I
G
N
L
P
S
I
E
S
G
K
H
S
G
L
K
W
H
Y
G
R
G
H
T
E
Q
Q
I
E
W
M
K
H
T
P
T
G
K
S
A
M1.HgaI
174
Y
K
K
S
L
K
W
D
M
P
K
S
Q
K
R
I
T
L
R
E
A
I
G
F
L
P
S
L
E
S
G
E
S
S
K
Y
K
W
H
Y
A
K
V
H
N
E
R
E
I
L
A
M
R
H
T
P
T
G
Q
S
A
M2.MhoVI
180
Y
K
Y
G
I
T
W
K
L
P
T
I
Q
A
E
I
S
L
Q
R
A
I
G
H
L
P
P
L
E
P
G
E
V
S
S
I
K
W
H
S
A
P
N
V
K
P
S
I
I
E
A
I
R
H
T
K
P
G
T
S
A
M2.HgaI
245
F
E
N
P
V
F
Y
P
K
N
E
I
T
G
E
R
L
K
G
Y
A
A
T
Y
K
R
M
D
W
D
K
P
A
P
T
I
T
M
R
N
D
C
I
S
S
Q
S
N
V
H
P
G
R
L
L
E
D
G
T
Y
M1.MhoVI
251
L
L
D
G
T
Y
M1.HgaI
F
E
N
L
V
H
Y
P
R
-
K
A
N
G
E
K
V
K
G
Y
H
S
S
Y
R
R
I
R
W
D
E
P
A
P
T
I
T
I
R
N
D
A
I
S
S
Q
R
N
V
H
P
G
R
P
235
M
K
N
E
V
Y
F
P
K
-
K
K
D
G
S
P
I
K
G
F
H
N
T
Y
K
R
M
K
W
D
E
P
C
P
A
R
A
M
N
N
G
N
M
G
G
H
N
N
V
H
P
G
R
P
L
E
N
G
T
Y
M2.MhoVI
241
I
S
N
P
I
F
Y
P
K
-
K
D
N
G
E
R
I
K
G
F
H
N
T
Y
K
R
M
E
W
D
K
P
A
P
A
R
T
T
Y
S
G
S
I
S
S
H
N
N
I
H
P
G
R
L
Q
L
D
G
T
Y
M2.HgaI
alpha helix
beta strand
identical AA
Supplementary Fig. S3: Phylogenetic analysis of HgaI-homologs.
(A) Order of conserved motifs of C5-MTases. Red = A-Adenosyl-L-methionin (SAM) binding motifs, green = catalytic motifs, black = traget recognition domain (TRD), gray = unassigned. (B-C) Phylogenetic trees including homologs of M1.HgaI (M1) and M2.HgaI (M2) considering the region I-X and only the TRD region, respectively. (D) Protein alignement of the TRD of MhoVI- and HgaI-MTases.
